# Vulnerability of Bangladeshi street-children to HIV/AIDS: a qualitative study

**DOI:** 10.1186/1471-2458-14-1151

**Published:** 2014-11-06

**Authors:** Md Jasim Uddin, Haribondhu Sarma, Tasnuva Wahed, Md Wazed Ali, Tracey Perez Koehlmoos, Quamrun Nahar, Tasnim Azim

**Affiliations:** Centre for Equity and Health Systems, icddr,b, Mohakhali, Dhaka, 1212 Bangladesh; Centre for Nutrition and Food Security, icddr,b, Mohakhali, Dhaka, 1212 Bangladesh; Centre for Vaccine Sciences, icddr,b, Mohakhali, Dhaka, 1212 Bangladesh; Centre for Population, Urbanization and Climate Change, icddr,b, Mohakhali, Dhaka, 1212 Bangladesh; Centre for HIV and AIDS, icddr,b, Mohakhali, Dhaka, 1212 Bangladesh

**Keywords:** Abuse, Risky behaviours, Street-children, Vulnerability, Bangladesh

## Abstract

**Background:**

Children living on the streets are an underprivileged population of Bangladesh and are likely to be more vulnerable to STIs/HIV for their day-to-day risky behaviours and lifestyles. This study assessed the vulnerability of Bangladeshi street-children to HIV/AIDS using qualitative participatory methods.

**Methods:**

This ethnographic participatory, qualitative study was conducted during February 2010– December 2011 among children aged 5–12 years, who live and/or work on the streets in Dhaka, the capital city of Bangladesh. Data were collected in three phases: (a) social mapping (n = 493), (b) participatory group discussions (n = 119), and (c) individual interviews (n = 36).

**Results:**

Results showed that street-children were engaged in behaviour that entails risk of exposure to HIV/AIDS. They possessed poor knowledge of the transmission of disease and of the benefits of using condoms; most of them reported never using a condom. The experience of selling sex for money and a variety of sexual activities, like anal, vaginal and oral sex, were commonly reported. The children also reported that they were regular users of one or more types of drugs, including those taken by injection.

**Conclusions:**

The deplorable living conditions of street children, with no obvious rights or way out, make them highly vulnerable to HIV/AIDS. Urgent attention of the policy- makers to implement services addressing issues relating to social conditions, sexual health, and drug-use is warranted to prevent the possible epidemic of HIV/AIDS among this group of population.

**Electronic supplementary material:**

The online version of this article (doi:10.1186/1471-2458-14-1151) contains supplementary material, which is available to authorized users.

## Background

The Asia-Pacific region is home to nearly half of the world’s children, including large numbers of street-children. Children aged 5–17 years constitute 32% of the total population (about 42 million) in Bangladesh, a poor country of South Asia. There are about 445,226 street-children in Bangladesh at present, and the number is continuously rising; of them, 75% live in the capital city Dhaka; 53% are boys and 47% are girls. It is projected by UNICEF that the number of street-children will grow to 1,615,330 by 2024 in Bangladesh [1]. In the cities, street-children are mostly found near railway stations, launch/boat terminals, bus stations, busy markets, commercial areas, parks/pavements, big mosques, and *mazar*s (mausoleum) [2–5]. About 45% of children live with parents while a good proportion of them live alone (22%) [6]. The causes of living on the street included poverty or hunger (89%), running-away from home (14%), torture by step mother/father (11%), earning money or income (11%), lack of guardians to look after (9%), and abuse (6%) [6, 7]. These children are usually deprived from basic human rights and have inadequate access to food, clothings, accommodation, education, and health facilities [8]. At very early ages, mostly at the age of 7 years, they get involved with different types of profession, such as helping hand of carpenters, hotel/restaurant boy, transport helper, household maid, shoe-shiner, and factory worker [9]. Although they work 6.5 hours per day, they earn only 45 BDT (1 US$ = 79 BDT) a day [6].

Although Bangladesh has a low prevalence (<1%) of HIV, data from the 9^th^ Round of HIV sero-surveillance showed that, over the 2000–2011 period, the trend in HIV infection is increasing [10]. Due to high HIV prevalence in neighboring countries, like India and Myanmar, bolstered with the presence of risk factors, like over population, poverty, migration, overindulgence of commercial and transactional sex, Bangladesh is to be considered a country with high vulnerability to HIV [11–14]. Children living on the streets, an underprivileged population of this country, are likely to be more vulnerable to STIs/HIV for their day-to-day risky behaviours and lifestyle. This vulnerability of the street-children to HIV/AIDS has been well-documented in many countries, particularly in Africa [15, 16]. For example, street-children in Ghana and India were found to be extremely vulnerable to STIs/HIV due to the high incidence of sexual abuse and exploitation [17, 18]. An Indian study reported that many street-children younger than eight years mentioned having sex for companionship or being victims of regular sexual abuse. They are typically faced with the risk of violence, which goes hand-in-hand with risks linked to drug-abuse and sexually transmitted infections (STIs), especially HIV [15, 19]. Life on the street for the girl children can be much more oppressive and exploitative than for boys: Girls aged 9–10 years are forced to consume drugs and are then sexually abused [18]. However, in Bangladesh, the vulnerability of street-children to HIV/AIDS has not yet been well-documented.

The methodology of obtaining information from street-children is a key factor in understanding the real situation of this highly-vulnerable group [20]. A study noted that “street-children involved in activities not approved by the society suppressed the nature of their work” [2]. Lying is commonly reported by researchers who work with street-children [21]. Traditional survey techniques tend to lead to results that reinforce traditional views, resulting in poor-quality data [20, 22]. Therefore, qualitative and participatory techniques have been recommended for the study of street-children [4, 21]. Moreover, researchers favour using ethnographic methods to highlight the subject’s perspective and to avoid overlooking important behavioural and situational factors.

This study analyzed the vulnerability of street-children to HIV/AIDS using ethnographic and qualitative methods for more nuanced understanding of their vulnerability to STIs/HIV. Evidence suggests that it is before the age of nine years that children have their first sexual experience that entail vulnerability to HIV. Evidence also suggests that sex workers who had sex before the age of 13 years reported more about symptoms of sexually transmitted infections (STIs) and more about drug-abuse [2]. Thus, this study explored the variation and multi-layered complexity of vulnerability of different groups of children, including both males and females who were living on the streets without family (abandoned), living on the street with family, and working on the street and returning to the family at night. This can help policy-makers and programme managers develop interventions specific to the needs of street-children.

## Methods

### Study design and population

This ethnographic, participatory, qualitative study was conducted during February 2010– December 2011 among children aged 5–12 years, who lived and/or worked on the street in the Dhaka city of Bangladesh. Data were collected between March 2010 and October 2010. Participants were selected purposively to ensure representation of both girls and boys in two age-groups (5–8 years and 9–12 years) in three categories of street-children, namely those who (a) slept on the street without families; (b) slept on the street with families; and (c) worked on the street but returned to their homes for sleeping.

### Activities for sampling and data collection

**Figure 1:**
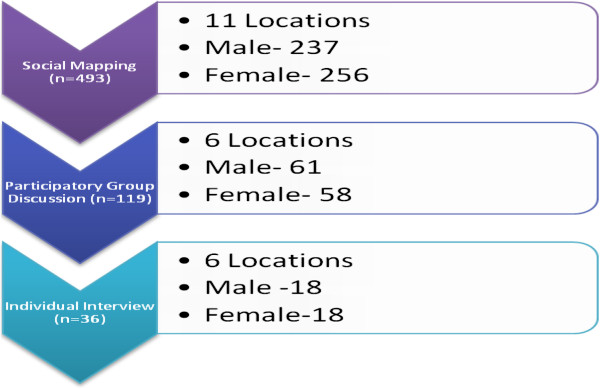
**Status of street-children who participated in different phases of the study.**

Study investigators recruited six field research officers (FROs) with prior experience of qualitative research and arranged intensive data-collection training. A research investigator was responsible for supervising their activities. Senior investigators met the team frequently to provide feedback to ensure quality. Three activities were then undertaken to collect data: (a) social mapping, (b) participatory group discussions (PGD), and (c) individual interviews (Figure 1).

Social mapping is an effective participatory tool for obtaining information about the community, its structure, noteworthy places, and social network of an area [23]. We conducted social mapping in 11 selected areas, consisting of five major points through which rural people enter Dhaka city and six locations with major concentrations of street-dwellers in order to identify street-children and their spots. In total, 66 social mapping sessions involving 493 street-children were conducted using a checklist. During social mapping, the interview team spent time in all the 11 study places/locations. They spent time walking with and talking to street-children where they live. To establish rapport and form trusted relationships with the children, the FROs participated in the children’s daily activities in their working areas and sleeping places. This informal involvement with them facilitated the easy flow of information and enabled them to share their personal stories with the FROs. Finally, FROs selected six spots (3 for boys and 3 for girls) from each of the study locations, based on the concentration of boys and girls, for each social mapping session. Social mapping took place at different times of the day, in the evening, and on different days of the week and attempted to capture the full range of activities of street-children in an area. They also enlisted 8–15 street-children from each spot to participate in the mapping session. For drawing a map, we selected silent and uninterrupted places (free from the presence of others) with the help of the participants, local potential persons (*vangari dokandars*, meaning dealers of scavenged valuables usually supplied by street-children), and NGO workers. We also used NGO-operated open air school places, storage rooms at the inter-district bus stations, offices of the associations of rickshaw-pullers, and separate rooms at shelter-homes run by NGOs where the street-children felt free to talk about all kinds of sensitive issues, such as sexual behaviour and use of drugs. On the day of drawing the social map, we organized the children, prepared the venue for drawing the map, took permission from the authorized person or organization, opened the art paper, and distributed colour pencils to them. We asked them questions according to the interview checklist and helped them to draw the map. We gave an orientation about what they would do in social mapping. Considering gender sensitivity, the male researchers conducted the social mapping session with male children and the female researchers with female children to ensure their openness to sensitive information. At every point, the social mapping sessions were conducted at different times, in different places, and on different days. The children fixed the time, place, and the day. We took assent from them and also consent from the parents/guardians of those children who lived with their parents on the street or returned to the family at night. The FROs wrote a transcript after the completion of each mapping session, and they subsequently prepared one report for each of the 11 areas. FROs prepared a session transcript and wrote a report for each of the 11 areas. Through initial analysis of these mapping transcripts, we selected six of the 11 locations for participatory group discussion (PGD) sessions and individual interviews where each location had a boundary encompassing an area with two-kilometre radius.

The PGDs were conducted with all-male or all-female groups of 3–5 children at a place of their choice. The children of the PGDs were selected based on their willingness to participate, experience of harassment, seeming to be more vulnerable, and freedom to share their experiences about their street-life. A PGD had three stages: at the first session, the children were asked to draw maps of places where they spend time; second, they drew pictures of a typical day, showing their work and leisure time, harassment, drug and sexual abuses, healthcare-seeking behaviour, knowledge of STIs/HIV/AIDS, and safer sex practices; in the final session, they identified key people in their personal networks with whom they had regular and important contacts. It required 2–3 hours for conducting a PGD session. During the PGD sessions, we had to give them break for some time whenever they felt weary because most of them were reluctant to sit for a long time in a seating. Since the street-children were dependant on their work, we arranged refreshments as compensation of their time. However, the time for conducting the PGD sessions was determined in consultation with the participants. In total, 36 PGD sessions (18 with boys and 18 with girls) with 119 street-children were conducted using a semi-structured guideline.

The team identified participants for individual interviews by analyzing PGD data. From each PGD, we selected one child purposively who had a range of vulnerable exposure to STIs/HIV, such as exposure to drugs and sex, living conditions, and geographical location. They used a flexible semi-structured interview guide on topics that are related to potential vulnerabilities, drug-abuse; sexual abuse; experiences, and healthcare-seeking behaviour. Thirty-six (18 girls and 18 boys) interviews were conducted, and field notes were written by interviewers. Interviews were not recorded because recording might inhibit truthful responses. Instead, the interviewers were trained to develop in-depth field notes following the interviews.

### Analysis of data

We used a thematic analysis for management and interpretation of data. The field team prepared and reviewed the transcripts daily and met with the investigators every second day to discuss their activity/interviews and for further guidance about note-taking and coding of transcripts. After reading, re-reading, and coding the text, the primary themes and sub-themes were merged with the main themes, using a matrix format (Additional file 1) where coded data were recorded systematically on a particular issue [24]. This matrix table facilitated display and interpretation of data. Denzin recommended triangulation of data considering different angles [25]. Accordingly, triangulation of data through different angles was employed to understand a particular issue (e.g. knowledge about STIs and HIV/AIDS) as recommended by Denzin [25].

### Ethical consideration

The research and ethical review boards of icddr,b (International Centre for Diarrhoeal Disease Research, Bangladesh) approved the study. Written consents or thumbprints from the children’s parents or guardians, if available, were taken. As parents or guardians of many children could not be traced, informed assents (collected written or thumbprint consents directly from the children) were collected from 305 children. FROs informed respondents properly and respectfully about the study and about their voluntary participation. They were ensured about their rights to withdraw from participation and that refusal would not lead to adverse consequences. Confidentiality of data was strictly maintained.

## Results

### Demographic characteristics

About half (256 boys, 237 girls) of the 493 children who participated in social mapping had never been to school. Almost one-third had been living on the street since birth. They had multiple occupations: scrap scavenging *(tokai*), begging, selling of items (hawker), sex work, stealing, and daily labour. Four children reported being involved in DIC (community-based Drop-in-Centre) activities. A DIC is an establishment designed to provide recreational, educational, or counseling services to a particular group, like street-population.

### Vulnerability to HIV/AIDs

Through analysis of data obtained from participatory group discussions and individual interviews, three main themes of vulnerability to HIV/AIDS were identified: (i) knowledge on STIs/HIV, (ii) risky and other behaviours, and (iii) living conditions.

### Knowledge on STIs/HIV

#### Knowledge about STIs and HIV/AIDS

Most street-children who participated in PGD displayed little knowledge and misperceptions concerning causes and transmission of STIs/AIDS. The majority did not even know the name of any sexual disease or its treatment. Although some girls reported that they knew symptoms of STIs, such as itching and soreness in the sexual organs, they did not know about the reasons of the soreness. Some children (12 of 119) thought that AIDS could be transmitted from one person to another by taking drugs together, washing kitchen materials with polluted water, having food in a single plate, or using a single tooth brush together. Thirty of 119 children had more sophisticated knowledge, saying that AIDS could be transmitted when people had sex with sex worker without a condom, when people received blood from a patient with HIV infection, or when children had anal sex with each other. Knowledge about STIs and HIV/AIDS was the highest (55%) among the abandoned male children who had access to the Drop in Center (DIC)-based services compared to other two categories.

#### Knowledge about condom-use

Poor knowledge regarding condom-use was common among all groups but the children who were living with their families or retuning home at night knew even less about it than children living on their own. They identified condoms as *potka* (balloons) and had seen those in parks, dustbins, drains, open fields, railway/bus stations, and shops. Most abandoned children were aware that condoms are used during sexual intercourse. A few of the abandoned children and of those living on the street with their families mentioned that condoms could prevent pregnancy and transmission of disease from one to another. They had learnt this information from their peers, sex workers, or a condom *apa* (a health worker who distributes condoms in their surroundings).

### Risky and other behaviours

#### Sexual behaviour

Nearly half of the children (48 of 119) participating in PGD sessions reported having sexual experience. They were involved with three types of sex: anal (32 of 119), vaginal (23 of 119), and oral (6 of 119). Although most children had their first experience as penetrative sex, a few had anal male-to-male sex with partners of their same age. Sometimes, in the absence of parents or guardians, children’s play included attempts at sexual intercourse or other sexual activities. In this regard, one of the abandoned children said:

*“The first time I had this experience was with one of my relatives. She was of my age. When she came to our sleeping place and our parents went out for work, other friends arranged my marriage with her. They also arranged bashor ghar (bride chamber/nuptial). In the bashor ghar, all friends left us alone, and we had sex (I tried to push my penis into her vagina but I could not) ....*” (An abandoned child, age: 11 years, sex: Male, no education, occupation: hawker)

#### Use of condom

None of the children with sexual experience reported using a condom, not even those who worked as sex workers. A girl said: *“The clients wanted to leave the semen directly on to the uterus, otherwise they did not get pleasure…….*” *(*An abandoned 12 year-old child with no education; occupation: beggar and sex worker; living on street for 3/4 years*).* A few male children said that they could not use it as the size of a condom is bigger than their penis.

#### Healthcare-seeking behaviour

A few female children reported that they had white discharge with bad smell, injury in the vagina and anus, with bleeding, itching, and soreness. Street-children reported being very reluctant to seek treatment for any health problems. Usually, they preferred discussing illness within their social network, such as with the peers or the buyers of things they scavenge. If they did not recover, they consulted local pharmacist, herbalist, or spiritualist (e.g. exorcising by *fakir*). They would visit a hospital or doctor only rarely when a problem became severe. However, hospital-based healthcare-seeking behaviour was totally absent among the abandoned girl children compared to the other two categories. One of the abandoned children expressed his coping mechanism during illness in the following way:

“*Once when I was attacked by high fever, the khala (a woman) who slept beside me on the street wrapped me tightly with a chador (cloth sheet), and next morning after taking a bread, I took Napa tablet and slept wrapping myself with the cloth sheet as it was suggested by an elder brother*.”

#### Drug-abuse

Data obtained through individual interviews showed that drug-use was common among almost all groups of street-children (27 of 36 respondents). Peers introduce them to *dandy* (glue sniffing) at first but they are gradually exposed to other drugs, such as *ganja* (marijuana)*, Bangla-mod* (locally-made wine), and *cakki* (sleeping pill) as well as heroin and other injectable drugs. Drug-use was lower among female children than among males.

In this regard, one male child said:

*“In the very first day, my peer (boy-friend) taught me how to smoke the left-out cigarette of passersby. First time the smoking made me phlegm but gradually I was adapted. One day when I went with a peer of different age beside a pond, the peer gave me a cigarette and encouraged me to smoke. It smelt bad but my peer strongly inspired me. I felt as if the lights poles were moving round, and I became senseless. Later on, I came to know from my peer that it was ganja, not cigarette.”*

#### Injecting drugs

Only abandoned children had the experiences of injecting drug-use, which was not common to other two categories of children. A total of three abandoned street-children (male = 2, female = 1) reported their addiction to injecting drugs. Sharing of needles among peers or friends was also reported by the children. One child stated:

*“At first I took ganja; after coming from home to Sadarghat I found so many boys taking ganja wrapped into the cigarette paper. I also took one or two sniffs with them and forgot about my home. After coming to Kamalapur station, I learned to take dandy and cakki from my working peers. My peers said that after taking cakki, people didn’t feel any pain if even a car went up the body or if someone beat. It keeps away from feeling of any smell if someone stays amidst the wastes. Moreover, after taking cakki, it makes a deep sleep; so, I take it. While scavenging scrap in Noabazar Bridge, Battikhana, Doaganj rail line, Chankharpul, Sadarghat, or Thathari bazaar, I found so many scavengers becoming addicted taking sui-injection. Bearing in mind those activities, one day when I expressed my wish to one of my working partners who took injecting drug, he gave me quarter mal-injection. After taking that it was a great pinic-addiction (addiction of these substances/drugs). After then, I only took injection instead of taking dandy, heroin, regularly.”*

### Living conditions

#### Mental harassment

All street-children in the participatory group discussions considered themselves a disgraced population. All reported feeling that people perceive them as thieves and swindlers. Adult scavengers, *vangari dokandars (*a vendor who purchase garbage/wastage/scraps things like plastics, metals, particles, glass from the scavengers and sales them for recycling*)*, and addicted men sometimes forcefully took or stole their collected scraps and/or often rebuked them if they brought fewer scraps. The children were also afraid of police vans, which might take them to the rehabilitation centres and of police threatening them while in a station. Illegal activities of their parents (e.g. drug-peddling, addiction, stealing, sex work) contributed to their harassment and mistreatment by others. In most cases, the children did not mention their own experiences but shared the experience of others. In this regard, one girl said:

*“I have a close friend whose father does not look after her. Because of financial hardship, her mother has to engage in illegal activities in brothel. But the people, who know it, tease my friend and use various abusive languages and also propose her to make physical relations with them. My friend is really an innocent girl but now she thinks to commit suicide because of this type of filthy talks.”*

The girls often face teasing and sexual solicitations by passersby, vegetable hawkers, drivers, rickshaw-pullers, and even other street-children.

#### Threats of violence

PGD data showed that a total of 36 boys and girls, representing all the groups of children, had been sexually harassed or raped. The perpetrators included adult street-dwellers, peers, employers, *mastans* (thuggish characters/violent persons), drivers/helpers of transports, guards/*ansars* (government internal security guards), brokers (pip), and shopkeepers, including NGO staff (from the community-based drop-in-centre). Victimization of rape was more prevalent among the older children (9–12 years) than the younger (5–8 years).

#### Activities at leisure time

Street-children liked to walk about and spent leisure time with peers in certain public places, such as the Zoo and Shishu (Children’s) Park. They sometimes climb onto the roofs of trains and travel to different railway stations. They are fond of watching movies and TV, and of playing video games. Enjoying *choti* (pornography) with friends (who can read) is a major pleasure of older children (9–12 years). A 12-year old boy who was living with his family in the street said:

*“If someone brings a nude magazine, everybody tries to keep it. We see the breast and vagina of the heroine. Our sona (penis) become hot and strong. One of our friends reads the story of fucking, and the remaining children listen to him* (A 12 years old boy who lives on the street with family for 2 years, 2 years of schooling, with scrap scavenging–‘*Tokai*’ as profession)”

#### Making friends through sharing blood

Making of *rokter bondhu* (friendship by sharing blood) is a common practice among all categories of the street-children. Two friends cut their hands or any exposed parts of the body, mix their blood, and promise to sacrifice and do everything for each other. In this regard, an 11-year old abandoned male street-child said,

“*I have no relatives, no parents, and no brothers. How can I survive when I become sick? My rokter bondhu is always with me.”*

## Discussion

This study sought diversity among different groups of children through multiple qualitative techniques but we found that all groups engage in behaviour that entails risk of exposure to HIV/AIDS and knew very little about the risk and how to reduce it. Their deplorable living conditions of street-children, with no obvious rights or way out, make them highly vulnerable to HIV/AIDS.

Scant knowledge of and misconceptions about HIV/AIDs among these street children are similar to what has been reported in Malawian, Pakistani, and Indian studies but differ from some African findings where, about half of the children knew that unprotected sex is the primary mode of HIV transmission [26–28]. An Indian study noted that talking about sex is a taboo, and access to sexual information is restricted in many cultures; this is why female street-children had doubts about sexually transmitted diseases [29, 30]. Although knowledge about condom-use was unsatisfactory in all groups, our study showed that children who were living with their families and returning home at night had even poorer knowledge about condoms than other (abandoned) children. Moreover, although a few children knew about condoms, none had ever used one during sexual intercourse. There is, thus, no preventive option in the street-children’s daily lives, putting them at risk of HIV.

The street-children are also vulnerable to HIV/AIDS because of the range of their sexual behaviours. They were not only victims of forced sex but were also involved in diverse sexual activities, including anal-vaginal-oral sex, either penetrative or non-penetrative. The high mobility of street-children and their multiple sex partners must also increase their risk of HIV, the finding being consistent with another study conducted by Milky [2]. The reported levels of drug addiction also contribute to vulnerability as has been noted in studies in Africa [16]. The existing NGO interventions for addicted children need to be reviewed to enhance their participation in these programmes. STI prevalence among street-children varies in different parts of the world from 6% to 36% [17, 29, 31].

Our study did not report any prevalence as no laboratory diagnosis was applied for detection. However, many children were reluctant to be tested, professed not to be worried about diseases, ignored formal healthcare, and appeared to be unaware of the risks and gravity of illnesses. An assessment of the existing healthcare services for street-children and their performance should be conducted promptly to address the challenges and increase the health service utilization practices by these children.

As in other studies [16, 28, 31], experience of violence and mental harassment were common among street-children in Dhaka city. These experiences were different among different categories of children, and the perpetrators represented a broad range of community members, including law-enforcing agents. Street-children enjoyed their free time with pornography. Lack of family support drives children to risky modes of survival, and they will even cut their own bodies to establish bonds of friendship to get support in emergency situations. To ameliorate their risky environments and behaviours, specific policy and interventions focusing on street-children are required in Bangladesh, which should be developed in a participatory way by engaging all the stakeholders, including the Ministry of Social Welfare. In addition, the present Vagabond Act and its implementation process need to be reviewed to make it child-friendly and to alleviate street-children’s fear of the vagabonds’ shelter-home. The environment of the shelter-homes should be improved with attention to health, hygiene, and children’s rights.

### Limitations

This study has some limitations. Our findings may not be generalized to the wider context due to its participatory nature and approaches that were applied to select street-children. The non-random recruitment of participants is prone to selection bias. Additionally, the results are based on the information given by children, who may have underreported because of the social stigma attached to sexual acts and consumption of intoxicating drugs. The results at best give an in-depth understanding about the vulnerability of street-children of Dhaka city to HIV/AIDS.

The findings identified a number of areas where policy-makers and programme personnel need to focus attention to reduce the vulnerability of street-children to HIV/AIDS in Dhaka city. The Ministry of Health and Family Welfare (MoHFW) and private sectors should come forward to ensuring future programmes to meet the needs of this extremely vulnerable group. Awareness-raising programmes for the children and the community, concerning risky behaviours, child rights, and child abuses, are essential to protect these children from ill-health and social deprivation. Programmes aimed at raising awareness, increasing knowledge and perception of risk about HIV/AIDS among children, their partners, gatekeepers, parents, and the community, need to be developed and implemented on an urgent basis to save this vulnerable group from infection. More specifically, comprehensive and targeted interventions, such as advocacy-based interventions, are needed for the gatekeepers of street-children, especially for *vangari dokandars* and adults living on street, by whom the children are mostly victimized. The advocacy should also target those groups (drivers and helpers of bus, truck, or CNG auto-rickshaw and peers of street-children) who sexually abuse street-children regularly. Finally, as the findings of the study indicate that street-children are a vulnerable group for HIV infections, they need to be included in the regular screening/surveillance process. These categories of children should also be included in the national serological survey so that, in the near future, more evidence-based information be available to help policy-makers in any future decision-making regarding interventions for street-children in the country.

## Conclusions

The study found that the street-children of Bangladesh are highly vulnerable to HIV. Urgent attention of the policy-makers is required to undertake and implement services addressing issues relating to social conditions, sexual health, and drug-abuse to prevent the possible epidemic of HIV among this group of population.

## Electronic supplementary material

Additional file 1: **Presentation of qualitative data with themes and sub-themes by category of children.** (DOC 80 KB)
