# Supplementary material for: Vulnerability of Bangladeshi street-children to HIV/AIDS: a qualitative study
Source: BMC Public Health. 2014 Nov 6;14:1151. doi: 10.1186/1471-2458-14-1151 (PMC4232676; doi:10.1186/1471-2458-14-1151)
Supplement: Supplementary file 1 — Additional file 1: Presentation of qualitative data with themes and sub-themes by category of children. (DOC 80 KB) [file 12889_2014_7229_MOESM1_ESM.doc]

**Additional file 1. Presentation of qualitative data with themes and sub-themes by category of children**

|  | **Abandoned children** | | **Children living on street with family** | | **Children working on street and returning to family at night** | |
| --- | --- | --- | --- | --- | --- | --- |
| **Theme 1: Knowledge on STIs/HIV** | | | | | | |
| ***Sub-theme: Knowledge about STIs and HIV/AIDS*** | | | | | | |
| Name of STDs | Itching, soreness | | Itching, soreness | | Itching | |
| Transmission | Through sex act, taking injecting drug, through mothers to child, through blood transfusion­, having food in a common plate,  using a common tooth, brush together, kissing | | Through sex act, taking injecting drug, washing kitchen materials with polluted water | | Through sex act, taking injecting drug, through mothers to child­ | |
| ***Sub-theme: Knowledge about condom-use*** | | | | | | |
| What is condom | Potka  Child’s playing materials | | Potka  Child’s playing materials | | Potka  Child’s playing materials | |
| Where available | Pharmacy ,shop, open fields | | Dustbins, drains, open fields, pharmacy/shop | | Pharmacy, dustbins,  drains, health worker (condom apa),  sex worker, shop | |
| Usefulness | Sexual intercourse, prevent pregnancy, prevent disease,  easy to have sex | | Sexual intercourse, prevent pregnancy, prevent disease,  easy to have sex | | Sexual intercourse, prevent pregnancy,  prevent disease | |
| Sources of information | Peers, health worker (Condom *Apa*)  DIC staff | | Peers, sex workers, health worker (Condom *Apa*) | | Peers, health worker (Condom *Apa*), DIC staff, relatives | |
| **Theme 2: Risky and other behaviours** | | | | | | |
| ***Sub-theme: Sexual behavior*** | | | | | | |
| Type of sex | Vaginal, anal, oral | | Vaginal, anal, oral | | Vaginal, anal, oral | |
| Sexual partners | Adult street people, peers,  sex worker/Hijra, street vendors, *koolis*, driver/helpers, rickshaw-pullers | | Adult street people, peers,  Hijra, sex workers, local mastan, relatives | | Peers, Hijra, street- vendors, drivers/helpers, sex workers, security guards, police | |
|  |  | |  | |  | |
| ***Sub-theme: Use of condom*** | | | | | | |
| Cause of not using condom | Less pleasure, bigger size | | Less pleasure, bigger size | | Less pleasure, bigger size | |
| ***Sub-theme: Healthcare-seeking behavior*** | | | | | | |
| Types of sickness that street children suffered | Fever/cold/cough, sore/ulcer,typhoid, dirrehoea, itching,  abdominal pain/headache,  jaundice ,conjunctivitis,  injured in anal and virginal route, asthma, dog bite, white discharge | | Fever/cold/cough,typhoid  sore/ulcer, dirrehoea,  itching, abdominal pain/headache, jaundice, anaemia | | Fever/cold/cough,  typhoid, sore/ulcer, dirrhoea, abdominal pain/headache, conjunctivitis | |
| Behaviour regarding healthcare- seeking | Did nothing (wait for natural cure) | | Did nothing (wait for natural cure) | | Did nothing (wait for natural cure) | |
| Places of treatment | Visited local pharmacies,  NGO clinic, hospital, icddr,b hospital | | Visited local pharmacies,  NGO clinic, hospital, icddr,b hospital,  traditional healers (herbalist,  spiritualist) | | Visited local pharmacies, NGO clinic, hospital, icddr,b hospital | |
| ***Sub-Theme: Drug abuse*** | | | | | | |
| Types of drug used by street- children | | *Dandy*, *Ganja*, *Chakki*, Heroin  Injection | | *Dandy*, *Ganja*, *Chakki* | | *Dandy*,  *Ganja*, *Chakki* |
| Way of being addicted | | Instigated by peers, friends, adult males, drivers/helpers, local big brothers | | Instigated by peers, friends, adult males, drivers/helpers, local big brothers | | Instigated by peers, friends, adult males, drivers/helpers, local big brothers |
| ***Sub-Theme: Injecting drugs*** | | | | | | |
| Way of being addicted | Peers/Friends | |  | |  | |
| Preferred part of body | Vein, muscles | |  | |  | |
| **Theme 3: Living conditions** | | | | | | |
| ***Sub-Theme: Mental harassment*** | | | | | | |
| Type of harassment | Mental, physical, sexual, economic | | Mental, physical, sexual, economic | | Mental, physical, sexual, economic | |
| Harassment by | Employers, police/guards, passersby, aged people,  peers, nastans, *vangari dokanders*, aged drug-addicted people, ricksha-pullers, DIC staff,  labourer (*kooli*), vegetable hawkers, drivers/helpers | | Employers, police/guards, passersby, aged people,  peers, nastans, *vangari dokanders*, aged drug-addicted people, ricksha-pullers, DIC staff,  labourer (*kooli*), vegetable hawkers, drivers/helpers | | Employers, police/guards, passersby, aged people,  peers, nastans, *vangari dokanders*, aged drug-addicted people, ricksha-pullers, DIC staff,  labourer (*kooli*), vegetable hawkers, drivers/helpers | |
| ***Sub-Theme: Threats of violence*** | | | | | | |
| Type of threats | Sexually harassed /raped | | Sexually harassed /Raped | | Sexually harassed /Raped | |
| Raped by | Adult street- dwellers, Peers  Employers, Mastans, Divers/helpers, Guards/Ansars  Brokers/Pip, Shopkeepers  NGO staff, Rickshaw puller | | Adult street- dwellers, Peers  Employers, Mastans, Divers/helpers, Guards/Ansars  Brokers/Pip, Shopkeepers  NGO staff, Rickshaw puller | | Adult street- dwellers, Peers  Employers, Mastans, Divers/helpers, Guards/Ansars  Brokers/Pip, Shopkeepers  NGO staff, Rickshaw puller | |
| ***Sub-Theme: Activities at leisure time*** | | | | | | |
| Spending leisure time with | Peers, adult males/females | | Peers | | Peers | |
| Leisure place | Airport, *mazar*, park, cinema hall, zoo , DIC, station, employer’s house | | *Mazar*, park, cinema hall,  zoo , station, stadium  DIC. lonely place, local shop | | *Mazar*, park. cinema hall, zoo . station,  video shop | |
| Activities during leisure | Watching movie,watching TV, singing/dancing, playng carom, loudu, video games, choti (pornography), taking drug, swimming | | Watching movie,watching TV, playng carom, loudu, video games, choti (pornography), taking drug,  games( Naikka muttia, cricket, boici, etc) | | Watching movie, Watching TV, games,  video games, choti (pornography), taking drug | |
